# Supplementary material for: OpenMM 7: Rapid development of high performance algorithms for molecular dynamics
Source: PLoS Comput Biol. 2017 Jul 26;13(7):e1005659. doi: 10.1371/journal.pcbi.1005659 (PMC5549999; doi:10.1371/journal.pcbi.1005659)
Supplement: S1 Source Code — (ZIP) [file pcbi.1005659.s002.zip › OpenMM-7.0.1-Source/docs/api-python/generated/simtk.openmm.app.simulation.Simulation.html]

Simulation — OpenMM 7.0.1.dev-5e86c4f documentation


# Simulation¶

*class* `simtk.openmm.app.simulation.``Simulation`(*topology*, *system*, *integrator*, *platform=None*, *platformProperties=None*, *state=None*)¶
:   Simulation provides a simplified API for running simulations with OpenMM and reporting results.

    A Simulation ties together various objects used for running a simulation: a Topology, System,
    Integrator, and Context. To use it, you provide the Topology, System, and Integrator, and it
    creates the Context automatically.

    Simulation also maintains a list of “reporter” objects that record or analyze data as the simulation
    runs, such as writing coordinates to files or displaying structures on the screen. For example,
    the following line will cause a file called “output.pdb” to be created, and a structure written to
    it every 1000 time steps:

    simulation.reporters.append(PDBReporter(‘output.pdb’, 1000))

    `__init__`(*topology*, *system*, *integrator*, *platform=None*, *platformProperties=None*, *state=None*)¶
    :   Create a Simulation.

        |  |  |
        | --- | --- |
        | Parameters: | - **topology** (*Topology*) – A Topology describing the the system to simulate - **system** (*System or XML file name*) – The OpenMM System object to simulate (or the name of an XML file   with a serialized System) - **integrator** (*Integrator or XML file name*) – The OpenMM Integrator to use for simulating the System (or the name   of an XML file with a serialized System) - **platform** (*Platform=None*) – If not None, the OpenMM Platform to use - **platformProperties** (*map=None*) – If not None, a set of platform-specific properties to pass to the   Context’s constructor - **state** (*XML file name=None*) – The name of an XML file containing a serialized State. If not None,   the information stored in state will be transferred to the generated   Simulation object. |

    Methods

    |  |  |
    | --- | --- |
    | `__init__`(topology, system, integrator[, ...]) | Create a Simulation. |
    | `loadCheckpoint`(file) | Load a checkpoint file that was created with saveCheckpoint(). |
    | `loadState`(file) | Load a State file that was created with saveState(). |
    | `minimizeEnergy`([tolerance, unit, maxIterations]) | Perform a local energy minimization on the system. |
    | `runForClockTime`(time[, checkpointFile, ...]) | Advance the simulation by integrating time steps until a fixed amount of clock time has elapsed. |
    | `saveCheckpoint`(file) | Save a checkpoint of the simulation to a file. |
    | `saveState`(file) | Save the current state of the simulation to a file. |
    | `step`(steps) | Advance the simulation by integrating a specified number of time steps. |

    `minimizeEnergy`(*tolerance=Quantity(value=10.000000000000004*, *unit=kilojoule/mole)*, *maxIterations=0*)¶
    :   Perform a local energy minimization on the system.

        |  |  |
        | --- | --- |
        | Parameters: | - **tolerance** (*energy=10\*kilojoules/mole*) – The energy tolerance to which the system should be minimized - **maxIterations** (*int=0*) – The maximum number of iterations to perform. If this is 0,   minimization is continued until the results converge without regard   to how many iterations it takes. |

    `step`(*steps*)¶
    :   Advance the simulation by integrating a specified number of time steps.

    `__delattr__`¶
    :   x.\_\_delattr\_\_(‘name’) <==> del x.name

    `__format__`()¶
    :   default object formatter

    `__getattribute__`¶
    :   x.\_\_getattribute\_\_(‘name’) <==> x.name

    `__hash__`¶

    `__reduce__`()¶
    :   helper for pickle

    `__reduce_ex__`()¶
    :   helper for pickle

    `__repr__`¶

    `__setattr__`¶
    :   x.\_\_setattr\_\_(‘name’, value) <==> x.name = value

    `__sizeof__`() → int¶
    :   size of object in memory, in bytes

    `__str__`¶

    `runForClockTime`(*time*, *checkpointFile=None*, *stateFile=None*, *checkpointInterval=None*)¶
    :   Advance the simulation by integrating time steps until a fixed amount of clock time has elapsed.

        This is useful when you have a limited amount of computer time available, and want to run the longest simulation
        possible in that time. This method will continue taking time steps until the specified clock time has elapsed,
        then return. It also can automatically write out a checkpoint and/or state file before returning, so you can
        later resume the simulation. Another option allows it to write checkpoints or states at regular intervals, so
        you can resume even if the simulation is interrupted before the time limit is reached.

        |  |  |
        | --- | --- |
        | Parameters: | - **time** (*time*) – the amount of time to run for. If no units are specified, it is   assumed to be a number of hours. - **checkpointFile** (*string or file=None*) – if specified, a checkpoint file will be written at the end of the   simulation (and optionally at regular intervals before then) by   passing this to saveCheckpoint(). - **stateFile** (*string or file=None*) – if specified, a state file will be written at the end of the   simulation (and optionally at regular intervals before then) by   passing this to saveState(). - **checkpointInterval** (*time=None*) – if specified, checkpoints and/or states will be written at regular   intervals during the simulation, in addition to writing a final   version at the end. If no units are specified, this is assumed to   be in hours. |

    `saveCheckpoint`(*file*)¶
    :   Save a checkpoint of the simulation to a file.

        The output is a binary file that contains a complete representation of the current state of the Simulation.
        It includes both publicly visible data such as the particle positions and velocities, and also internal data
        such as the states of random number generators. Reloading the checkpoint will put the Simulation back into
        precisely the same state it had before, so it can be exactly continued.

        A checkpoint file is highly specific to the Simulation it was created from. It can only be loaded into
        another Simulation that has an identical System, uses the same Platform and OpenMM version, and is running on
        identical hardware. If you need a more portable way to resume simulations, consider using saveState() instead.

        |  |  |
        | --- | --- |
        | Parameters: | **file** (*string or file*) – a File-like object to write the checkpoint to, or alternatively a filename |

    `loadCheckpoint`(*file*)¶
    :   Load a checkpoint file that was created with saveCheckpoint().

        |  |  |
        | --- | --- |
        | Parameters: | **file** (*string or file*) – a File-like object to load the checkpoint from, or alternatively a filename |

    `saveState`(*file*)¶
    :   Save the current state of the simulation to a file.

        The output is an XML file containing a serialized State object. It includes all publicly visible data,
        including positions, velocities, and parameters. Reloading the State will put the Simulation back into
        approximately the same state it had before.

        Unlike saveCheckpoint(), this does not store internal data such as the states of random number generators.
        Therefore, you should not expect the following trajectory to be identical to what would have been produced
        with the original Simulation. On the other hand, this means it is portable across different Platforms or
        hardware.

        |  |  |
        | --- | --- |
        | Parameters: | **file** (*string or file*) – a File-like object to write the state to, or alternatively a filename |

    `loadState`(*file*)¶
    :   Load a State file that was created with saveState().

        |  |  |
        | --- | --- |
        | Parameters: | **file** (*string or file*) – a File-like object to load the state from, or alternatively a filename |

High performance molecular simulation on GPUs


### Navigation

- Application Layer
  - Loaders and Setup
  - Representation and Manipulation
  - Simulation
  - Reporting Output
  - Extras
- Library Layer

©2015, Stanford University and the Authors.
|
Powered by Sphinx 1.3.1
& Alabaster 0.7.7
|
Page source
